# Supplementary material for: Curiosity shapes spatial exploration and cognitive map formation in humans
Source: Commun Psychol. 2024 Dec 30;2:129. doi: 10.1038/s44271-024-00174-6 (PMC11685098; doi:10.1038/s44271-024-00174-6)
Supplement: Supplementary file 2 — Supplementary Information [file 44271_2024_174_MOESM2_ESM.pdf]

Supplementary Information for

Curiosity shapes spatial exploration and cognitive map formation in humans

Danlu Cen[1], Eva Teicher[t1], Carl J. Hodgetts[1,2], Matthias J. Gruber[1]
[1] Cardiff University Brain Research Imaging Centre (CUBRIC), School of Psychology, Cardiff University, Wales, UK.
[2] Department of Psychology, Royal Holloway, University of London, London, UK.

Corresponding author: Danlu Cen (danlu.cen@outlook.com)

The file includes:

Supplementary Methods
Supplementary Note 1: Correlation between path RE and head-direction RE
Supplementary Note 2: Correlation between curiosity ratings and interest ratings
Supplementary Note 3: Interest-visual exploration relationship after controlling for room type
Supplementary Note 4: Curiosity, interest, exploration and cognitive map formation at inter- and intra-individual levels
Supplementary Note 5: Follow-up analysis on cognitive map formation dimensions

Supplementary Figures 1-9
- Supplementary Figure 1
- Supplementary Figure 2
- Supplementary Figure 3
- Supplementary Figure 4
- Supplementary Figure 5
- Supplementary Figure 6
- Supplementary Figure 7
- Supplementary Figure 8
- Supplementary Figure 9

Supplementary References

Supplementary Methods

Virtual stimuli

The study utilised a virtual environment created in Unity 3D (version 2019.4.15, Unity Technologies), comprising an invariant outdoor setting and various indoor scenes (i.e., virtual rooms) that differed across trials. The outdoor area featured a pier connected to the virtual room by a zigzag-shaped pathway. Participants began each trial on the pier, navigating through the pathway to enter a room for exploration.

Eighteen distinct rooms were created, with two designated for familiarisation and practice (see Fig. S1 for snapshots of these rooms). All the rooms were uniform in virtual dimensions ($16m \times 16m$ in virtual space). In order to enhance the rooms' realism and encourage participants' exploration, we deliberately clustered the room with furniture and decorations. The furniture and decorations in the rooms were downloaded from online 3D asset stores (ArchVizPRO) and then imported into the virtual room in Unity 3D. In each trial, room types (e.g., Museum, Library, Office) were displayed above the entrance, as illustrated in Figure 1A. www.3d66.com) and then imported into the virtual room in Unity 3D. In each trial, room types (e.g., Museum, Library, Office) were displayed above the entrance, as illustrated in Figure 1A.

We also included a total of 156 3D models of everyday objects, sourced from online 3D model stores (www.sketchfab.com and www.turbosquid.com), which served as stimuli for an incidental memory test. Of these objects, 108 were strategically placed along the pathway (12 for practice and 96 during exploration), while 48 acted as lures in the memory tests. The presentation order and categorization of these objects were randomised for each participant. Large-scale items, such as aeroplanes, were excluded to maintain realism.

Detailed procedures

The experiment was structured into three distinct phases: familiarisation, exploration, and memory tests. In the main study, participants commenced with the Five-Dimensional Curiosity Revised scale (5DCR) [1] prior to the familiarisation phase. The exploration phase closely followed the familiarisation phase, and the memory tests took place either approximately 24 hours later (Experiment 1) or immediately after the exploration phase (Experiment 2).

Familiarisation Phase: This phase comprised three trials, designed to acquaint participants with navigating the virtual environment and the experimental procedures. The first trial, in which participants traversed through an emptied pathway and then visited an empty room, served as a practice for movement controls within the virtual world. In the subsequent two trials, participants explored one of two rooms (Cinema and Bridal Shop, order counterbalanced; see Fig. S1A). In these two trials, the procedure was the same as in the exploration phase (see below), to help participants familiarise themselves with the experimental flow.

Exploration Phase: Participants visited 16 rooms (Fig. S1B) in a randomised order. Each trial began at the pier, where participants could view the room's label on its front wall (illustrated in Fig. 1B). Upon seeing the label, a question about their level of curiosity towards the room appeared, which participants rated on a Likert scale from 1 ("*Not curious at all*") to 10 ("*Very much curious*"), by inputting their response as an integer number using the keyboard (see Fig. 1B for the interface).

As participants walked along the zigzag-shaped pathway to the room, they would encounter six objects placed at intervals in the corners, each initially hidden by a pathway fence and only visible upon approach. An invisible constraint ensured participants could only move normally when facing the object ahead, standardising view duration across objects, trials and participants. While these objects were described as irrelevant to the task, participants were instructed not to stop but to continue walking past them. As they passed each object, it disappeared with a "cling" sound, aiding in encoding these incidental stimuli.

Upon reaching the room, participants opened the door by pressing "E" on the keyboard, experiencing a 5-second door opening animation. Inside, participants were instructed to explore each room freely without any

time constraints and allowed to engage with the environment at their own pace. Participants' position in the room (location data) and angle of field of view (head direction data) were recorded at a screen frame rate of 60 Hz. When deciding to leave, participants pressed "B", leading to another rating display where they rated how interested they felt about the room on a Likert scale from 1 ("Not interesting at all") to 10 ("Very much interesting").

Memory test in Experiment 1

In Experiment 1, participants were invited to return to the lab approximately 24 hours after completing the initial exploration task to finish the second part of the experiment. In this part of the experiment, participants took part in a surprise memory test. This test focused on the recognition memory of objects encountered on the pathway outside each room. Participants were presented with a randomised series of 144 images on the computer screen, comprising 96 objects they had previously seen (6 per room) and 48 novel objects (lures). They responded to each object by pressing designated keys to indicate if the object was "remembered" (key 1), "familiar" (key 2) or "new" (key 3). The recognition memory test was self-paced without a time limit for each trial. As this surprise memory test assessed participants' memory for incidentally encountered objects on the pathway outside the rooms, which was not relevant to our primary research questions in this manuscript around spatial exploration and cognitive map formation of the rooms themselves, the results of this test are not reported here.

Memory test in Experiment 2

In contrast to Experiment 1, Experiment 2 implemented immediate memory tests following a brief break of approximately 5 minutes after the exploration phase. These tests were designed to assess memory of room details and participants' introspective experiences during exploration.

Sketch map task and free recall: The first two memory tests were integrated into a single procedure. Participants were asked to sketch the layout of each explored room and subsequently describe the visual details they recalled. The rooms in these tasks were presented in a randomised order, differing from their exploration sequence. To ensure uniformity in detail recalling and map drawing, we standardised the instructions and provided participants with examples for the two rooms presented in the familiarisation phase.

At the onset of each trial, participants viewed an image of the room's label (e.g., Bedroom, Lounge or Classroom), similar to those seen during exploration. The sketching task required drawing the room's layout on a provided paper sheet, marked with the room's type and an outlined square representing the room's boundaries. Participants were encouraged to include spatial elements like furniture, doors and windows. Example sketches of the Bridal Shop and Cinema, explored during familiarisation, were shown for guidance.

Following the sketch map task, participants described the room's visual details (e.g., colours, shapes, sizes, number of objects, positions, lighting) in a text input field on the screen. This memory test was mainly focused on the memory of the items and visual details of the room, which contains little spatial aspects of memory. Even where there was spatial information, it largely overlapped with the spatial memory assessed in the drawing test. Therefore, these text responses were not included in the current study due to the focus on cognitive map formation and to avoid introducing additional complexity that could detract from our primary research objectives.

Introspective Recall: The third test presented participants with recordings of themselves exploring the rooms. While watching, they were asked to recall and report any thoughts or feelings experienced during the original exploration. Participants had the option to pause the video by pressing the spacebar, which would bring up a text input pop-up window for them to record their responses. Importantly, if the participant did not initiate a response within 7 seconds, the text input pop-up window would automatically appear, serving as a

prompt for them to engage in recall. However, the 7-second response interval was not disclosed to the participants, ensuring their reactions remained as spontaneous and natural as possible. The order in which the rooms appeared in the recordings was randomised, differing from both their original exploration sequence and the order in the previous memory tests. Again, findings from this introspective recall test were not included in the current study due to its focus on exploration and cognitive map formation.

Supplementary Note 1: Correlation between path roaming entropy and head-direction roaming entropy

We examined the correlation between path roaming entropy and head-direction roaming entropy by devising a Bayesian multivariate multilevel model with both path roaming entropy and head-direction roaming entropy as the two outcomes and the model includes no predictors except intercept. We investigated the correlation for both the intercept and residuals. The former reflects the correlation between path roaming entropy and head-direction roaming entropy across participants, and the latter reflects the correlation within participants. That is, a positive intercept correlation indicates that participants with a high average path roaming entropy tend to have a high average head-direction roaming entropy as well. On the other hand, a positive residual correlation indicates that participants who have a higher path roaming entropy than expected (for some rooms) also tend to have a higher head-direction roaming entropy (for these rooms).

In Experiment 1, the posterior mean of the intercept correlation between path roaming entropy and head-direction roaming entropy was 0.79, with a 93% HPDI of [0.64, 0.92]. The posterior mean of residual correlation was 0.40, with a 93% HPDI of [0.32, 0.47]. In Experiment 2, the posterior mean of the intercept correlation between path roaming entropy and head-direction roaming entropy was 0.75, with a 93% HPDI of [0.64, 0.86]. The posterior mean of the residual correlation was 0.44, with a 93% HPDI of [0.39, 0.49]. The findings suggest that path roaming entropy and head-direction roaming entropy positively correlate at the between- and within-subjects level, which highlights that our main analysis approach was appropriate to account for these correlations.

Furthermore, as shown in the main text, we also examined the residual correlation between path roaming entropy and head-direction roaming entropy after accounting for the predictors in the model. This further confirms the correlation between the two exploratory behaviours.

In two separate models, we examined whether the relationship between path roaming entropy and head-direction roaming entropy depends on the duration of time spent inside the room, respectively for Experiments 1 and 2. In these models, head-direction roaming entropy was included as the outcome, path roaming entropy, duration and their interaction were included as predictors. The models revealed that the relationship between path roaming entropy and head-direction roaming entropy was negatively impacted by the duration of time inside the room (Experiment 1: posterior mean of β = -0.0078, 93% HPDI = [-0.01, -0.0056]; Experiment 2: posterior mean of β = -0.0022, 93% HPDI = [-0.0037, -0.0008]). These results indicated that the longer the participant was inside the room, the weaker the relationship between the path roaming entropy and head-direction roaming entropy of their exploration. This could imply that when the duration is longer, participants may spend more time standing still and observing their surroundings or specific objects, rather than walking and observing simultaneously, leading to a decreased correlation between the two roaming entropy values.

Supplementary Note 2: Correlation between curiosity ratings and interest ratings

We examined the relationship between curiosity ratings and interest ratings in a similar manner. In Experiment 1, the posterior mean of the intercept correlation between curiosity ratings and interest ratings was 0.44, with a 93% HPDI of [-0.02, 0.87]. The posterior mean of the residual correlation was 0.55, with a 93% HPDI of [0.49, 0.61]. In Experiment 2, the posterior mean of the intercept correlation between curiosity ratings and interest ratings was 0.78, with a 93% HPDI of [0.60, .94]. The posterior mean of the residual correlation was 0.53, with a 93% HPDI of [0.48, 0.57]. The results indicated a positive correlation between curiosity ratings and interest ratings.

Supplementary Note 3: Interest-visual exploration relationship after controlling for room type

The interest ratings were acquired after the exploration phase. Environmental factors, such as the number or type of objects in the room, could plausibly influence both visual exploration behaviour (as measured by head-direction roaming entropy) and perceptions of room interestingness. This raises the possibility that the observed relationship between interest ratings and visual exploration could be confounded by these environmental factors. To address this, we included room type as an additional covariate in our model of head-direction roaming entropy, alongside curiosity, interest ratings, and time spent in each room. The inclusion of room type helps control for potential environmental confounds, such as object density or other features that may affect both exploration behaviour and interest ratings. We used the default flat prior for room type in the 'brms' package, reflecting a lack of strong assumptions about its effect.

Despite accounting for room type, interest ratings remained positively associated with head-direction roaming entropy in both experiments (Experiment 1: posterior mean = 0.0023, 93%-HPDI = [-0.0011, 0.0058]; Experiment 2: posterior mean = 0.0034, 93%-HPDI = [0.0008, 0.0064]). In Experiment 1, the uncertainty was higher, with a wider distribution, likely due to the smaller sample size. To further verify these results, we combined the data from both experiments and refitted the model. The positive relationship between interest ratings and head-direction roaming entropy persisted in this combined analysis (posterior mean = 0.0030, 93%-HPDI = [0.0007, 0.0051]). The posterior distributions for the relationship between interest rating and head-direction roaming entropy after controlling for toom type are plotted in Fig. S7.

Supplementary Note 4: Curiosity, interest, exploration and cognitive map formation at inter- and intra-individual levels

To further explore the impact of state curiosity, interest, spatial exploration (path roaming entropy), and visual exploration (head-direction roaming entropy) on the precision of cognitive map formation at both inter- and intra-individual levels, we computed a Bayesian multilevel model on the composite memory score. The model included pre-room curiosity, post-room interest, path roaming entropy, and Head-direction roaming entropy as predictors, with duration as a covariate. Notably, each predictor was split into two components: one representing the individual average centred around the grand mean across all participants (inter-individual level), and the other reflecting the trial-level value centred around the individual mean (intra-individual level). Random effects for the intra-individual predictors were nested within participants.

At the intra-individual level (reported in the main text but for completeness of the full analysis also included here), the precision of cognitive map formation was positively associated with pre-room curiosity (posterior mean = 0.066, 93%-HPDI = [0.027, 0.10]) and path roaming entropy (posterior mean = 0.80, 93%-HPDI = [0.008, 1.6]). There was also a potential negative association with post-room interest (posterior mean = -0.023, 93%-HPDI = [-0.060, 0.014]), while the relationship with Head-direction roaming entropy was small and uncertain (posterior mean = -0.075, 93%-HPDI = [-1.22, 1.12]).

At the inter-individual level, the model did not provide strong evidence for an association between the average precision of cognitive map formation and average pre-room curiosity (posterior mean = 0.04, 93%-HPDI = [-0.23, 0.14]), average post-room interest (posterior mean = -0.018, 93%-HPDI = [-0.019, 0.15]), or average path roaming entropy (posterior mean = 0.63, 93%-HPDI = [-1.04, 2.36]). However, there was some evidence of a positive association with average Head-direction roaming entropy (posterior mean = 2.22, 93%-HPDI = [-0.47, 4.80]), suggesting that participants who engaged in more visual exploration tended to form better cognitive maps on average.

To investigate whether the positive relationships between cognitive map precision and pre-room curiosity and path roaming entropy at the intra-individual level might be influenced by baseline performance, we examined the correlation between the random intercepts and the random slopes for pre-room curiosity and path roaming entropy. We found a potential negative correlation between the random intercepts and pre-room curiosity random slopes (posterior mean = -0.36, 93%-HPDI = [-0.79, 0.066]), indicating that participants with higher baseline performance showed a weaker effect of curiosity on cognitive map precision. There was also a small positive correlation between the random intercepts and path roaming entropy random slopes (posterior mean = 0.22, 93%-HPDI = [-0.30, 0.77]), though the evidence for this association was weak.

Supplementary Note 5: Follow-up analysis on cognitive map formation dimensions

We conducted detailed analyses to explore the effects of pre-room curiosity, post-room interest, path roaming entropy and head-direction roaming entropy on each of the four dimensions of cognitive map formation: Object Presence (OP), Spatial Distortion and Rotation of Features (SD), Relative Positioning (RP) and Spatial Proportion (SP). Separate Bayesian multilevel models were computed for each dimension as the outcome variable. For OP, the model utilised the complete dataset. For SD, RP and SP, in contrast, we excluded trials where the OP score was 1, indicating that none of the layout-defining objects in the room was accurately recalled. Each model incorporated pre-room curiosity, post-room interest, path roaming entropy and head-direction roaming entropy as predictors, with varying effects nested within participants.

Pre-room curiosity demonstrated a positive effect on OP (posterior mean = 0.084, 93%-HPDI = [0.049, 0.12]), SD (posterior mean = 0.064, 93%-HPDI = [0.013, 0.11]) and SP (posterior mean = 0.069, 93%-HPDI = [0.020, 0.12]), with a possible positive effect on RP (posterior mean = 0.056, 93%-HPDI = [-0.0047, 0.12]) (see Fig. S8, first column).

Post-room interest showed a negative effect on OP (posterior mean = -0.037, 93%-HPDI = [-0.072, 0.0034]), SD (posterior mean = -0.048, 93%-HPDI = [-0.10, 0.0052]), and RP (posterior mean = -0.034, 93%-HPDI = [-0.094, 0.027]), with negative but uncertain effect on SP (posterior mean = -0.014, 93%-HPDI = [-0.058, 0.034]) (see Fig. S8, second column).

Path roaming entropy showed positive effects on OP (posterior mean = 1.01, 93%-HPDI = [0.29, 1.74]), potentially positive effects on SD (posterior mean = 0.88, 93%-HPDI = [-0.24, 1.96]), and positive but uncertain effects on RP (posterior mean = 0.33, 93%-HPDI = [-0.93, 1.65]) and SP (posterior mean = 0.26, 93%-HPDI = [-0.88, 1.37]) (see Fig. S8, third column).

Head-direction roaming entropy showed no clear effect on OP (posterior mean = -0.20, 93%-HPDI = [-1.34, 0.98]), SD (posterior mean = 0.38, 93%-HPDI = [-1.21, 1.98]), RP (posterior mean = 0.072, 93%-HPDI = [-1.68, 1.88]), or SP (posterior mean = 0.29, 93%-HPDI = [-1.11, 1.77]) (see Fig. S8, fourth column).

Detailed findings are visualised in Fig. S8, with each column corresponding to the effects of the respective predictors on the cognitive map formation dimensions.

Supplementary Figure 1

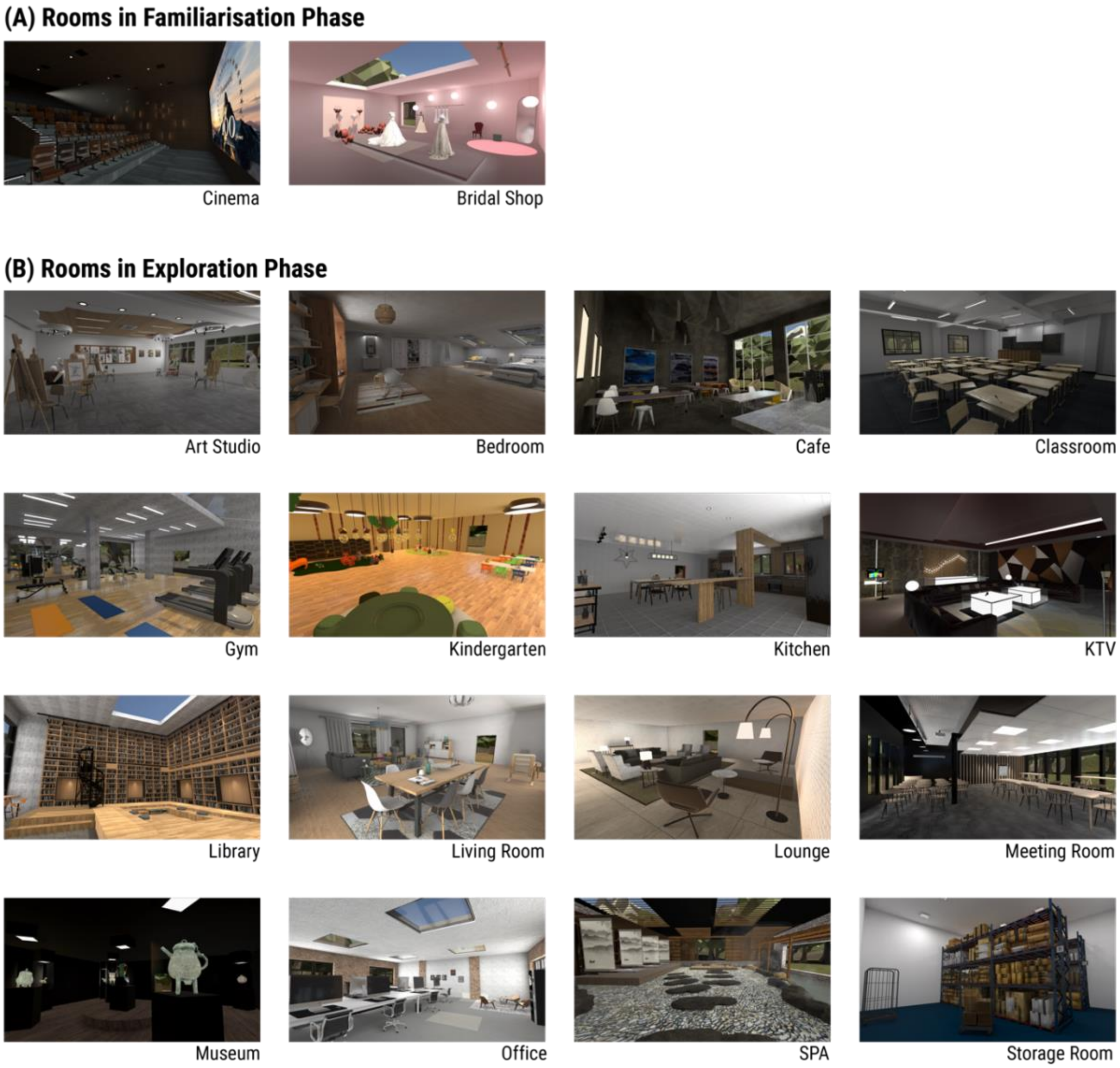

Figure S1. (A) Screenshots of the two rooms in the familiarisation phase: Cinema (left) and Bridal Shop (right). (B) Screenshots of the rooms in the exploration phase: Art Studio, Bedroom, Café, Classroom, Gym, Kindergarten, Kitchen, Karaoke (KTV), Library, Living Room, Lounge, Meeting Room, Museum, Office, SPA and Storage Room.

Supplementary Figure 2

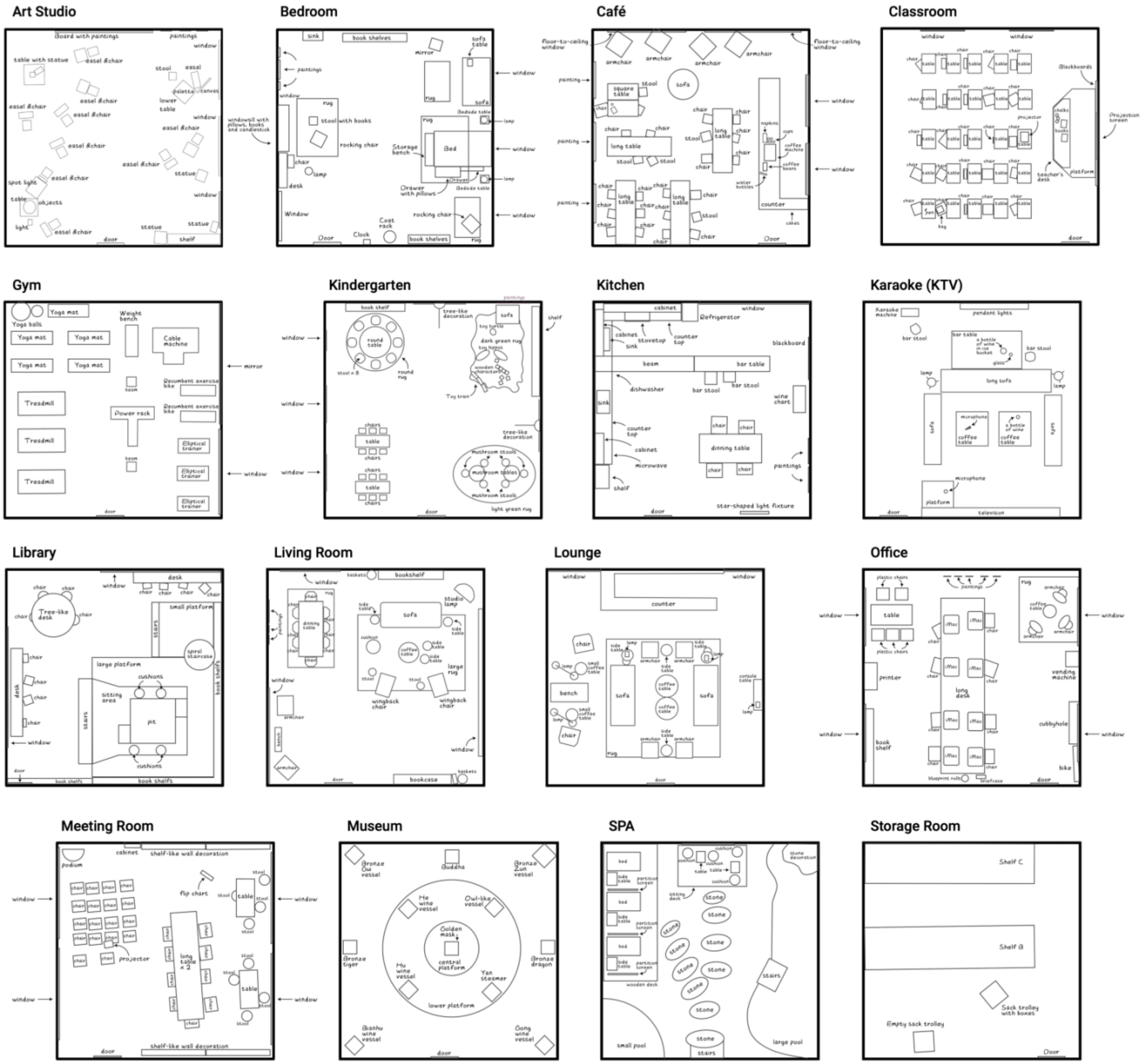

Figure S2. Layout of the rooms in the exploration phase: Art Studio, Bedroom, Café, Classroom, Gym, Kindergarten, Kitchen, Karaoke (KTV), Library, Living Room, Lounge, Meeting Room, Museum, Office, SPA and Storage Room.

Supplementary Figure 3

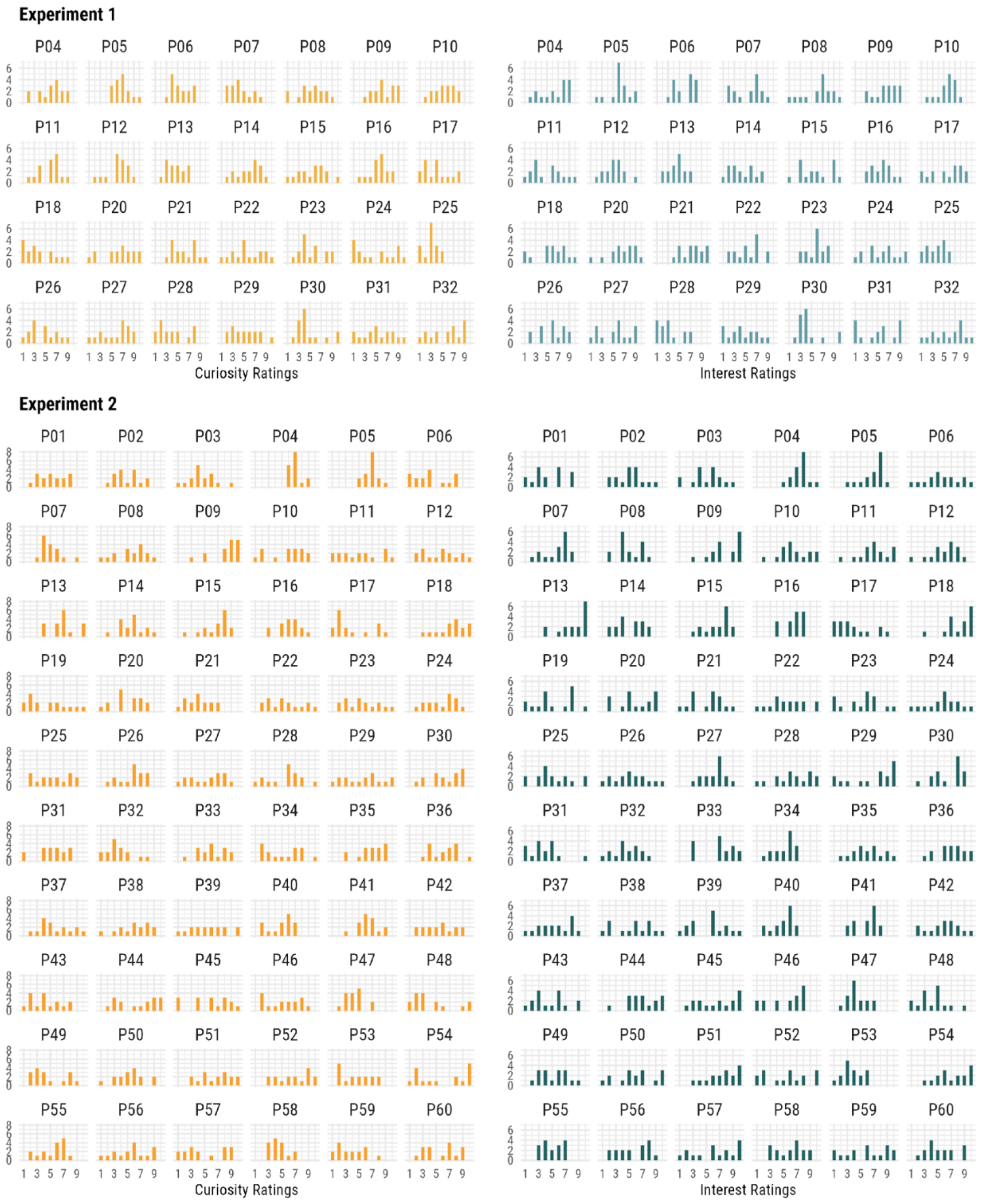

Figure S3. Distributions of curiosity ratings (left column) and interest ratings (right column) per participant for Experiment 1 (upper panel) and Experiment 2 (lower panel).

Supplementary Figure 4

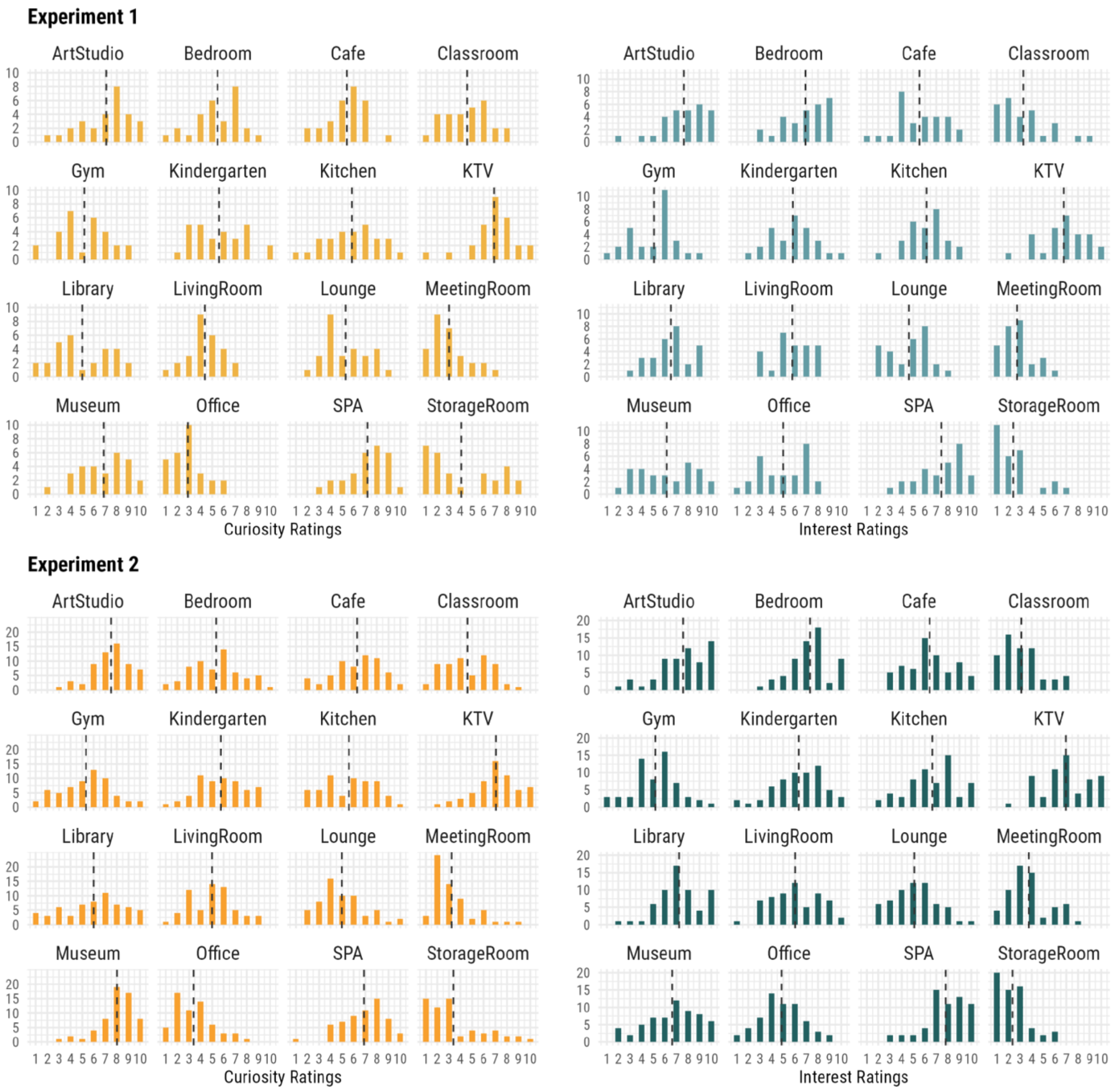

Figure S4. Distributions of curiosity ratings (left column) and interest ratings (right column) for each room in Experiment 1 (upper panel) and Experiment 2 (lower panel). Vertical dashed lines represent the average rating for the rooms.

Supplementary Figure 5

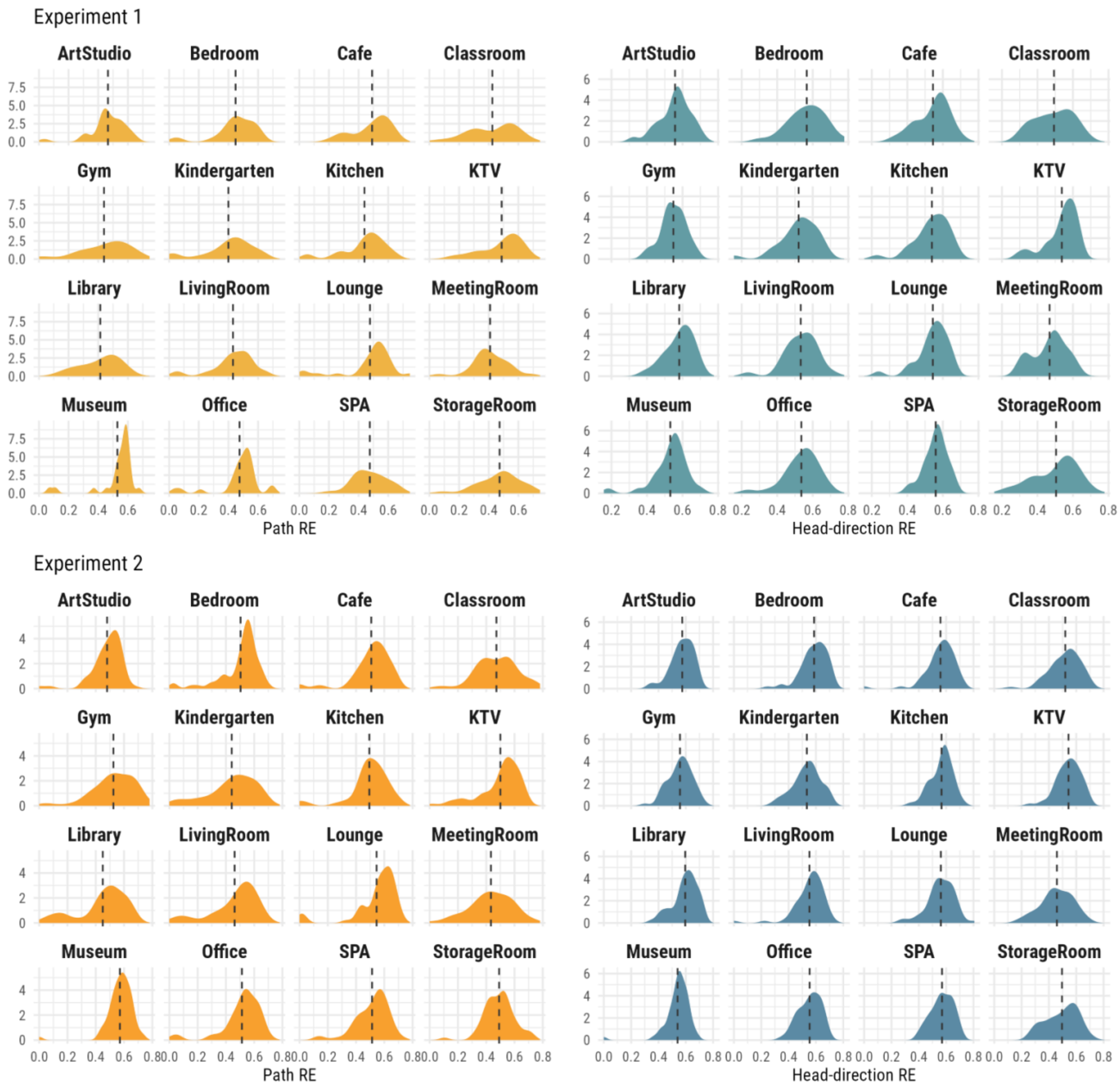

Figure S5. Distributions of path roaming entropy (Path RE) (left column) and head-direction roaming entropy (Head-direction RE) (right column) for each room in Experiment 1 (upper panel) and Experiment 2 (lower panel). Vertical dashed lines represent the average path roaming entropy or head-direction roaming entropy for the rooms.

Supplementary Figure 6

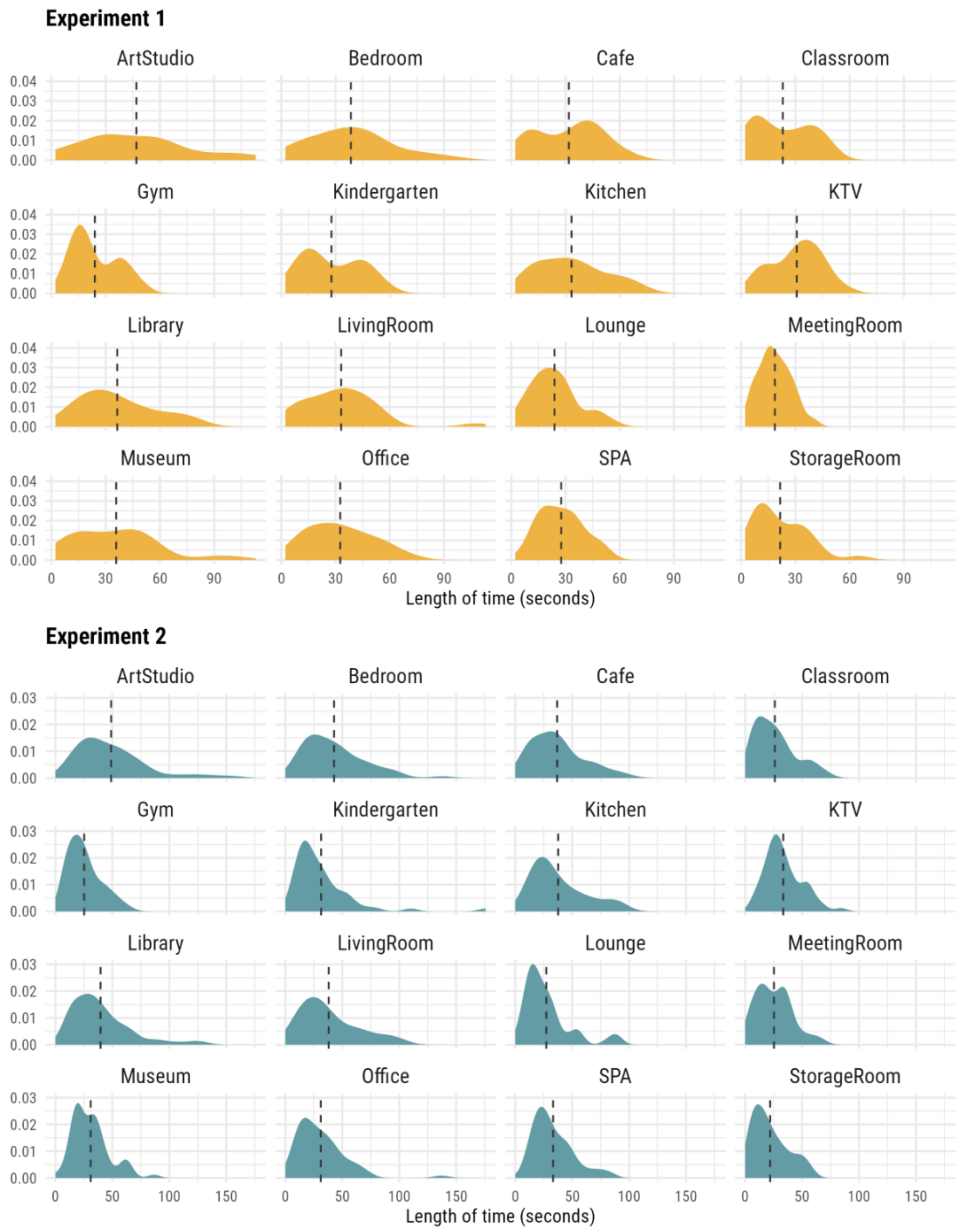

Figure S6. Distribution of length of time spent in each room for Experiment 1 (upper panel) and Experiment 2 (lower panel). Vertical dashed lines represent average duration that participants spent in the rooms.

Supplementary Figure 7

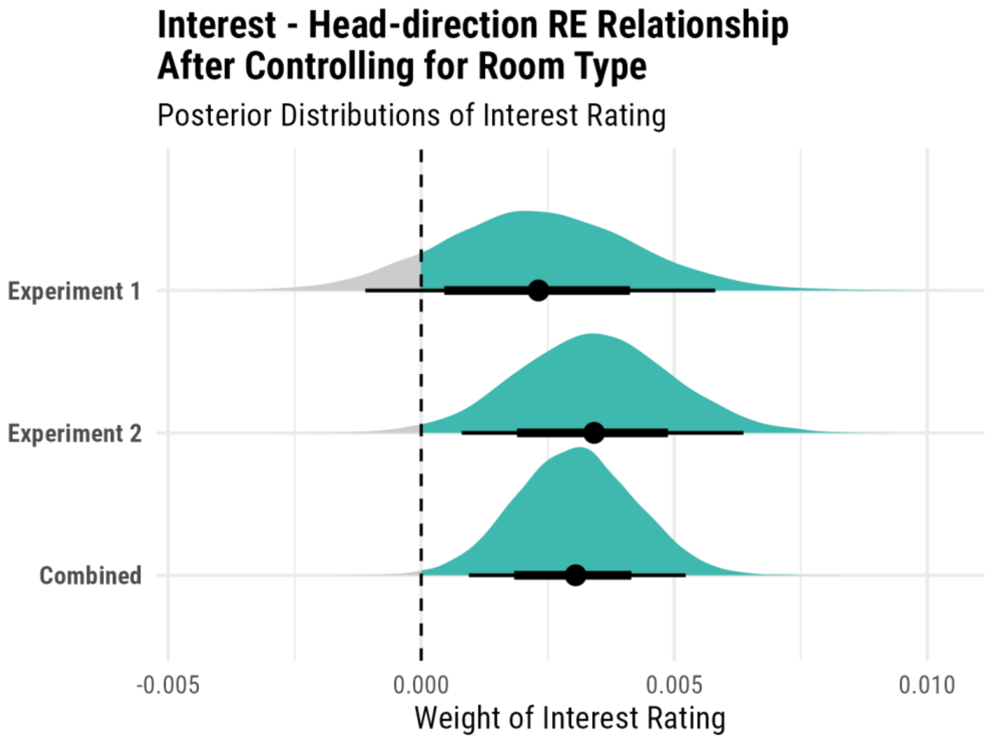

Figure S7: Posterior weight distributions for interest ratings on head-direction roaming entropy, while controlling for room type. Shown for Experiment 1 (top), Experiment 2 (middle), and both experiments combined (bottom). Green shading indicates the probability of a positive relationship between interest rating and head-direction roaming entropy, while the grey overlap represents the probability of a negative relationship. The black dot denotes the mean of the posterior distribution, indicating its central tendency. Horizontal bars represent the 67% (thick) and 93% (thin) highest probability density intervals (HPDIs). The vertical dashed line at zero indicates no relationship.

Supplementary Figure 8

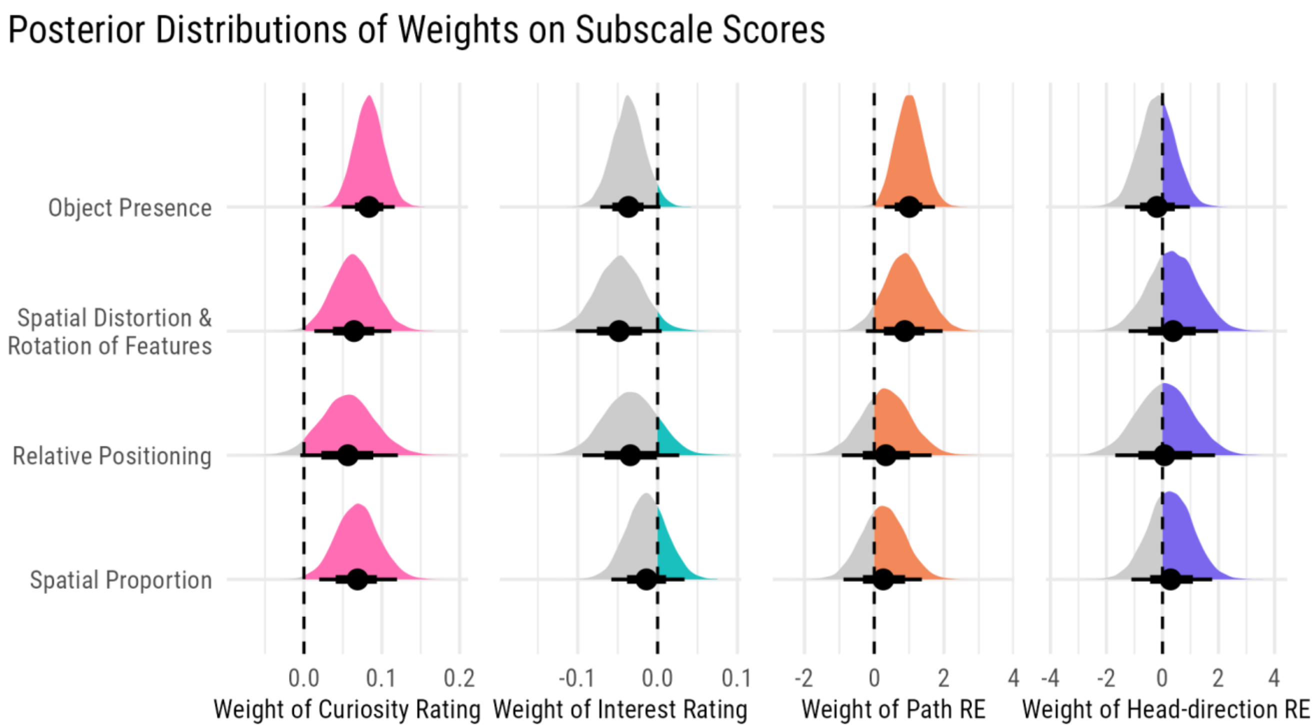

Figure S8. Posterior distributions of weights of four predictors on the cognitive map formations scores across four dimensions. From left to right, the columns represent pre-room curiosity rating, post-room interest rating, path roaming entropy (Path RE) and head-direction roaming entropy (Head-direction RE). Each row, from top to bottom, corresponds to the dimensions of cognitive map formation: Object Presence, Spatial Distortion and Rotation of Features, Relative Positioning and Spatial Proportion. The distributions illustrate the degree to which each predictor influences the dimension score, with the width of the distribution indicating the level of uncertainty in the estimate. Black dots represent the means of the posterior distributions. Horizontal bars indicate the 67% (thick) and 93% (thin) HPDIs, quantifying the uncertainty of the estimates. Dashed vertical lines at zero point indicate a null effect of the predictor on the dimension score.

Supplementary Figure 9

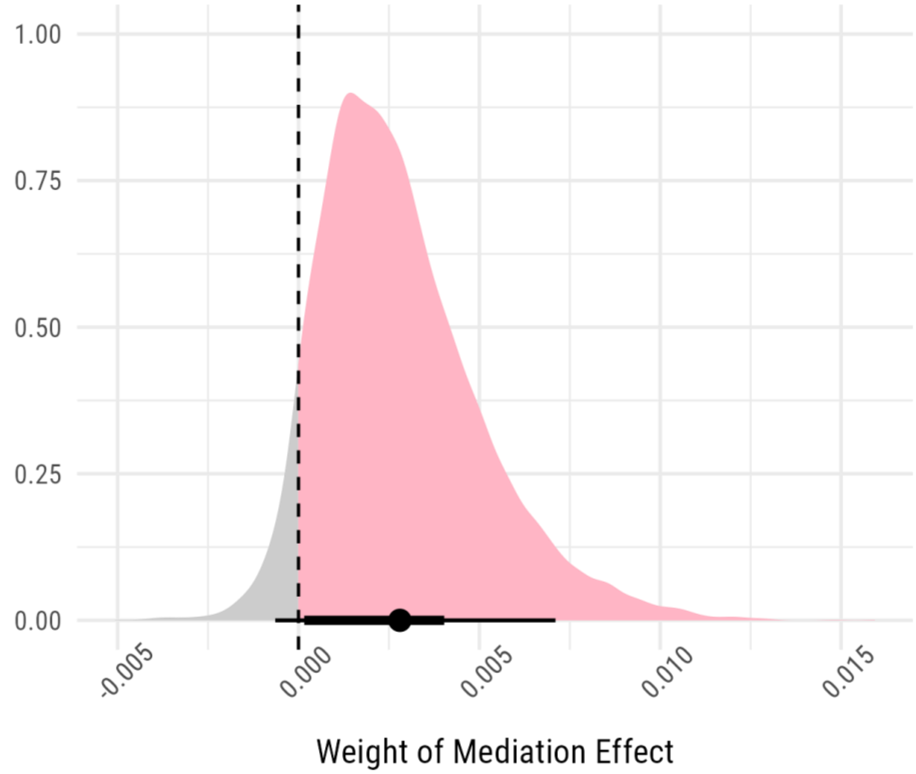

Figure S9. Posterior distribution of path roaming entropy mediation effect on cognitive map formation. The posterior distribution is illustrated for the mediation effect of path roaming entropy on the relationship between pre-room curiosity and cognitive map formation. The pink shaded area represents the probability density of positive mediation effects, while the overlapping grey area reflects the probability density of negative mediation effects. Black dot denotes the mean of the posterior distribution, serving as an indicator of the distribution's central tendency. Horizontal bars represent the 67% (thick) and 93% (thin) HPDIs. Dashed vertical line at zero point indicates the threshold for a null mediation effect.

Supplementary References

1. Kashdan, T. B., Disabato, D. J., Goodman, F. R. & McKnight, P. E. The Five-Dimensional Curiosity Scale Revised (5DCR): Briefer subscales while separating overt and covert social curiosity. *Pers. Individ. Dif.* **157**, 109836 (2020).
