# Supplementary material for: Curiosity shapes spatial exploration and cognitive map formation in humans
Source: Commun Psychol. 2024 Dec 30;2:129. doi: 10.1038/s44271-024-00174-6 (PMC11685098; doi:10.1038/s44271-024-00174-6)
Supplement: Supplementary file 3 — Reporting Summary [file 44271_2024_174_MOESM3_ESM.pdf]

nature portfolio

Corresponding author(s): Danlu Cen (danlu.cen@outlook.com)

Last updated by author(s): Nov 14, 2024

Reporting Summary

Nature Portfolio wishes to improve the reproducibility of the work that we publish. This form provides structure for consistency and transparency in reporting. For further information on Nature Portfolio policies, see our Editorial Policies and the Editorial Policy Checklist.

Statistics

For all statistical analyses, confirm that the following items are present in the figure legend, table legend, main text, or Methods section.

n/a	Confirmed	
☐	☒	The exact sample size (*n*) for each experimental group/condition, given as a discrete number and unit of measurement
☐	☒	A statement on whether measurements were taken from distinct samples or whether the same sample was measured repeatedly
☐	☒	The statistical test(s) used AND whether they are one- or two-sided<br>*Only common tests should be described solely by name; describe more complex techniques in the Methods section.*
☐	☒	A description of all covariates tested
☐	☒	A description of any assumptions or corrections, such as tests of normality and adjustment for multiple comparisons
☐	☒	A full description of the statistical parameters including central tendency (e.g. means) or other basic estimates (e.g. regression coefficient) AND variation (e.g. standard deviation) or associated estimates of uncertainty (e.g. confidence intervals)
☒	☐	For null hypothesis testing, the test statistic (e.g. *F*, *t*, *r*) with confidence intervals, effect sizes, degrees of freedom and *P* value noted<br>*Give P values as exact values whenever suitable.*
☐	☒	For Bayesian analysis, information on the choice of priors and Markov chain Monte Carlo settings
☐	☒	For hierarchical and complex designs, identification of the appropriate level for tests and full reporting of outcomes
☒	☐	Estimates of effect sizes (e.g. Cohen's *d*, Pearson's *r*), indicating how they were calculated

Our web collection on statistics for biologists contains articles on many of the points above.

Software and code

Policy information about availability of computer code

Data collection	The data were collected via a customized desktop program developed using Unity 3D (version 2019.4.15, Unity Technologies, Copenhagen Denmark). The exterior environment (e.g., hills, water, pier and pathway) and the basic structure of the virtual rooms (e.g., walls, floor and ceiling) were constructed in Unity 3D.The furniture and decorations in the rooms were downloaded from online 3D asset stores (ArchVizPRO Interior packages and www.3d66.com) and then imported into the virtual room in Unity 3D. The virtual objects displayed on the pathway were 3D models of everyday objects, sourced from online 3D model stores (www.sketchfab.com and www.turbosquid.com).<br><br>The precision scores of cognitive map formation on the participants' drawings were collected via an online coding platform developed in house (https://map-scoring.vercel.app/). This platform was designed to aid raters in efficiently coding the layout maps that participants drew in the memory test. It also allowed the raters to systematically manage their scoring records. This platform also offered a transparent and easily navigable workspace for raters to reconcile discrepancies. The platform is programmed via Next.js. The code that construct the platform can be accessed at https://osf.io/s2ja7/.
Data analysis	Data were analyzed with Python (3) and R (4.3.2) scripts. The code allowing to reproduce the presented analyses is available on OSF (https://osf.io/sc37a/).

For manuscripts utilizing custom algorithms or software that are central to the research but not yet described in published literature, software must be made available to editors and reviewers. We strongly encourage code deposition in a community repository (e.g. GitHub). See the Nature Portfolio guidelines for submitting code & software for further information.

Data

Policy information about availability of data

All manuscripts must include a data availability statement. This statement should provide the following information, where applicable:

- Accession codes, unique identifiers, or web links for publicly available datasets
- A description of any restrictions on data availability
- For clinical datasets or third party data, please ensure that the statement adheres to our policy

All data with preprocessed roaming entropy values is available at https://osf.io/sc37a/.

Research involving human participants, their data, or biological material

Policy information about studies with human participants or human data. See also policy information about sex, gender (identity/presentation), and sexual orientation and race, ethnicity and racism.

Reporting on sex and gender	We gathered demographic information through a preliminary questionnaire given prior to the experimental tasks. Participants were inquired about their age and gender, allowing them to self-identify their gender. Across both experiments, we had a total of 92 participants, with 32 in Experiment 1 and 60 in Experiment 2. The gender distribution was predominantly women, with 90.2% identifying as women (Experiment 1: n=28; Experiment 2: n=55) and 9.8% as men (Experiment 1: n=4; Experiment 2: n=5).<br><br>We did not conduct sex- and gender-based analyses in our research. This decision was grounded in the lack of prior evidence suggesting an impact of gender on the dynamics between curiosity, spatial exploration, and cognitive map formation. Consequently, our study was not designed with hypotheses specific to sex and gender differences in these contexts.<br><br>Consent for sharing individual-level data, including disaggregated sex and gender information, was obtained as part of our study's ethical considerations. However, due to the absence of sex- and gender-based hypotheses, detailed disaggregation beyond the overall gender distribution is not applicable to our findings.
Reporting on race, ethnicity, or other socially relevant groupings	We collected demographic data through a brief questionnaire administered to participants prior to their participation in the experiments. The questionnaire solicited information on gender and age (in years) only. We did not inquire about race, ethnicity, or other socially relevant groupings as these variables were not directly pertinent to our research questions. Furthermore, in our analyses, we did not control for age and gender, as our hypotheses did not revolve around the influence of these variables on the relationship between curiosity, spatial exploration, and cognitive map formation. Our decision was based on a focused examination of curiosity and spatial behaviors without presupposing the influence of these demographic factors.
Population characteristics	Demographic data in both experiments shows that participants' ages ranged between 18-25 years (Experiment 1 and Experiment 2), 90.2% were women (9.8% men).
Recruitment	Participants were recruited from Cardiff University through the Experimental Management System (EMS) of the School of Psychology. Our recruitment criteria included individuals with self-reported normal hearing and normal or corrected-to-normal vision to ensure that all participants could clearly perceive the experiment stimuli. However, this approach may introduce a self-selection bias, as it primarily attracts participants who are already engaged with or interested in psychological research, potentially limiting the generalizability of our findings to broader populations. Additionally, by recruiting from a university setting, our sample may lean towards a younger demographic with higher education levels than the general population. These factors should be considered when interpreting the results, as they may influence the relationship between curiosity, spatial exploration, and cognitive map formation.
Ethics oversight	The ethical committee of the School of Psychology at Cardiff University

Note that full information on the approval of the study protocol must also be provided in the manuscript.

Field-specific reporting

Please select the one below that is the best fit for your research. If you are not sure, read the appropriate sections before making your selection.

☐ Life sciences ☒ Behavioural & social sciences ☐ Ecological, evolutionary & environmental sciences

For a reference copy of the document with all sections, see nature.com/documents/nr-reporting-summary-flat.pdf

Behavioural & social sciences study design

All studies must disclose on these points even when the disclosure is negative.

Study description	This is a quantitative study in which participants completed cognitive tasks on desktop computers in lab.

Research sample	Our study sample comprised Cardiff University students recruited via the School of Psychology's Experimental Management System (EMS). In Experiment 1, we had 32 participants (average age = 19.75, SD = 1.63; 87.5% women and in Experiment 2, 60 participants (average age = 19.6, SD = 1.28; 91.7% women This sample, while convenient for our study's context, is not representative of the general population, primarily reflecting a younger demographic with a higher proportion of women.
Sampling strategy	Our study employed a convenience sampling strategy, recruiting participants from Cardiff University's student population via the School of Psychology's Experimental Management System (EMS). Given the novel aspects of our study, there were no existing studies to guide a sample-size calculation based on similar experimental designs or effect sizes. Therefore, our sample sizes for Experiment 1 (N=32) and Experiment 2 (N=60) were determined based on pragmatic considerations, including the availability of participants and the logistical capacity to manage the study within our time frame. We aimed for a minimum of 60 participants for Experiment 2 to ensure sufficient power for individual difference analyses, drawing on our previous research experience and commonly accepted practices in psychological research for studies of this nature.
Data collection	The data collection process was executed using desktop PCs, with visual stimuli displayed on LCD monitors (1920×1080 resolution; 60 Hz refresh rate). These sessions took place in the cognitive labs at the Cardiff University Brain Research Imaging Centre (CUBRIC), designed to facilitate controlled experimental conditions. During each session, only the participant and a single researcher were present in the lab to minimize distractions and maintain the integrity of the data collection process. The researcher was not blinded to the experimental conditions or the study hypotheses, which could potentially introduce bias; however, the structured nature of the experimental design aimed to mitigate this risk.
Timing	Experiment 1: January 2020 to February 2020; Experiment 2: February 2023 to March 2023
Data exclusions	In Experiment 1, a total of four participants' data were excluded from the analyses. The initial three participants were excluded due to adjustments made to the scales used for measuring curiosity and interest after their participation. Additionally, one participant was excluded due to incomplete exploration data. This participant engaged with only four of the 16 rooms designed for the experiment, which was insufficient for our analysis criteria. These exclusion criteria were determined post hoc to ensure the integrity and consistency of the dataset used for our final analysis.<br><br>In Experiment 2, data from five participants in the memory test were excluded from the relevant analysis because we slightly altered the memory test procedure after their participation. This adjustment necessitated their exclusion to ensure consistency and reliability in the analysis of the memory test results.
Non-participation	No participants dropped out/declined participation.
Randomization	Participants were not allocated into experimental groups.

Reporting for specific materials, systems and methods

We require information from authors about some types of materials, experimental systems and methods used in many studies. Here, indicate whether each material, system or method listed is relevant to your study. If you are not sure if a list item applies to your research, read the appropriate section before selecting a response.

Materials & experimental systems

n/a	Involved in the study
☒	☐ Antibodies
☒	☐ Eukaryotic cell lines
☒	☐ Palaeontology and archaeology
☒	☐ Animals and other organisms
☒	☐ Clinical data
☒	☐ Dual use research of concern
☒	☐ Plants

Methods

n/a	Involved in the study
☒	☐ ChIP-seq
☒	☐ Flow cytometry
☒	☐ MRI-based neuroimaging

Plants

Seed stocks	*Report on the source of all seed stocks or other plant material used. If applicable, state the seed stock centre and catalogue number. If plant specimens were collected from the field, describe the collection location, date and sampling procedures.*
Novel plant genotypes	*Describe the methods by which all novel plant genotypes were produced. This includes those generated by transgenic approaches, gene editing, chemical/radiation-based mutagenesis and hybridization. For transgenic lines, describe the transformation method, the number of independent lines analyzed and the generation upon which experiments were performed. For gene-edited lines, describe the editor used, the endogenous sequence targeted for editing, the targeting guide RNA sequence (if applicable) and how the editor was applied.*
Authentication	*Describe any authentication procedures for each seed stock used or novel genotype generated. Describe any experiments used to assess the effect of a mutation and, where applicable, how potential secondary effects (e.g. second site T-DNA insertions, mosiacism, off-target gene editing) were examined.*
